# Evolutionary and Ecological Characterization of Mayaro Virus Strains Isolated during an Outbreak, Venezuela, 2010

**DOI:** 10.3201/eid2110.141660

**Published:** 2015-10

**Authors:** Albert J. Auguste, Jonathan Liria, Naomi L. Forrester, Dileyvic Giambalvo, Maria Moncada, Kanya C. Long, Dulce Morón, Nuris de Manzione, Robert B. Tesh, Eric S. Halsey, Tadeusz J. Kochel, Rosa Hernandez, Juan-Carlos Navarro, Scott C. Weaver

**Affiliations:** Author affiliations: University of Texas Medical Branch, Galveston, Texas, USA (A.J. Auguste, N.L. Forrester, K.C. Long, R.B. Tesh, S.C. Weaver);; Universidad de Carabobo, Naguanagua-Valencia, Venezuela (J. Liria);; Ciudad Universitaria, Caracas, Venezuela (D. Giambalvo, M. Moncada, D. Morón, R. Hernandez);; Centro de Investigaciones de Virosis Hemorrágicas y Enfermedades Transmisibles, Guanare, Portuguesa State, Venezuela (N. de Manzione);; US Naval Medical Research Unit No. 6, Lima, Peru (E.S. Halsey, T.J. Kochel);; Universidad Central de Venezuela, Caracas (J.-C. Navarro)

**Keywords:** Mayaro virus, alphavirus, viruses, epidemic, outbreak, phylogeny, evolution, ecology, coalescent analysis, niche modeling, zoonoses, Venezuela

## Abstract

In 2010, an outbreak of febrile illness with arthralgic manifestations was detected at La Estación village, Portuguesa State, Venezuela. The etiologic agent was determined to be Mayaro virus (MAYV), a reemerging South American alphavirus. A total of 77 cases was reported and 19 were confirmed as seropositive. MAYV was isolated from acute-phase serum samples from 6 symptomatic patients. We sequenced 27 complete genomes representing the full spectrum of MAYV genetic diversity, which facilitated detection of a new genotype, designated N. Phylogenetic analysis of genomic sequences indicated that etiologic strains from Venezuela belong to genotype D. Results indicate that MAYV is highly conserved genetically, showing ≈17% nucleotide divergence across all 3 genotypes and 4% among genotype D strains in the most variable genes. Coalescent analyses suggested genotypes D and L diverged ≈150 years ago and genotype diverged N ≈250 years ago. This virus commonly infects persons residing near enzootic transmission foci because of anthropogenic incursions.

## Abstract 2

This virus commonly infects persons residing near enzootic transmission foci because of anthropogenic incursions.

The family *Togaviridae*, genus *Alphavirus*, includes several major mosquito-borne pathogens. Alphaviruses can be categorized as Old or New World viruses, which are generally associated with febrile illnesses with either arthralgic or encephalitic syndromes, respectively (*1**,**2*). Mayaro virus (MAYV), an exceptional arthralgic New World alphavirus, produces Mayaro fever, which has signs and symptoms similar to those of dengue fever, including an acute febrile illness of 3–5 days’ duration, typically with headache, retro-orbital pain, arthralgia, myalgia, vomiting, diarrhea, and rashes (*3**,**4*). Previous work has shown that some patients have persistent, severe joint pains for up to 1 year (*5*).

MAYV has a single-strand, positive-sense RNA genome ≈11,700 nt in length. The first two thirds of the genome encodes 4 nonstructural proteins (NSP1–4) and the other one third encodes the structural proteins (capsid, envelope [E] 3, E2, 6K/TF, and E1). Previous phylogenetic studies using a fragment of the structural polyprotein open reading frame suggest that MAYV occurs in 2 distinct genotypes, D and L (*6*). Genotype D includes isolates from all countries where MAYV has been detected, and genotype L contains strains detected only in Brazil (*6*).

MAYV was first isolated from forest workers in Mayaro, Trinidad in 1954 (*7*). Since 1954, there have been sporadic outbreaks of Mayaro fever (*5**,**6**,**8**–**11*), but most have occurred in Brazil, with the exception of a small outbreak in Bolivia in 2007 with 12 reported cases. MAYV has been isolated or antibodies against the virus were detected in Brazil, Colombia, Ecuador, Peru, Surinam, Bolivia, French Guiana, and Trinidad (*12**–**19*). Human MAYV infections were also detected serologically in Venezuela in a family that spent a night within a forest area (*20*).

The MAYV enzootic transmission cycle is not fully characterized. Previous studies suggest that it circulates between canopy-dwelling mosquitoes of the genus *Haemagogus* and nonhuman primates (*6**,**21*). Consequently, MAYV human seropositivity is largely associated with forest workers and hunters (*18*). MAYV has the potential to cause large outbreaks, as demonstrated in Brazil, where ≈800 persons were affected (*10*). Furthermore, *Aedes aegypti* mosquitoes are moderately competent vectors (*22*), which suggests that an urban human–mosquito–human transmission cycle could emerge, as has occurred for dengue, chikungunya, and yellow fever viruses with similar enzootic forest cycles (*23*).

In January 2010, an outbreak of Mayaro fever in Venezuela occurred in La Estación village, Portuguesa State. By June 4, a total of 77 cases were recorded, which represents one of the largest outbreaks detected in South America. To understand the origins, evolution, and ecoepidemiology of MAYV, we sequenced complete genomes of 6 strains isolated during this outbreak and 21 additional isolates, which represented the full known spectrum of MAYV genetic diversity. We performed robust phylogenetic analyses on our complete genome data and previously published partial E2–E1 sequences.

## Materials and Methods

### Study Site and Outbreak Details

The study protocol was approved by the Naval Medical Research Center and Naval Medical Research Unit No. 6 Institutional Review Boards (protocols NMRCD.2000.0006, 2010.0010, and NAMRU6.2012.0016). Approval was given in compliance with all applicable federal regulations governing the protection of human subjects.

**Figure 1:**
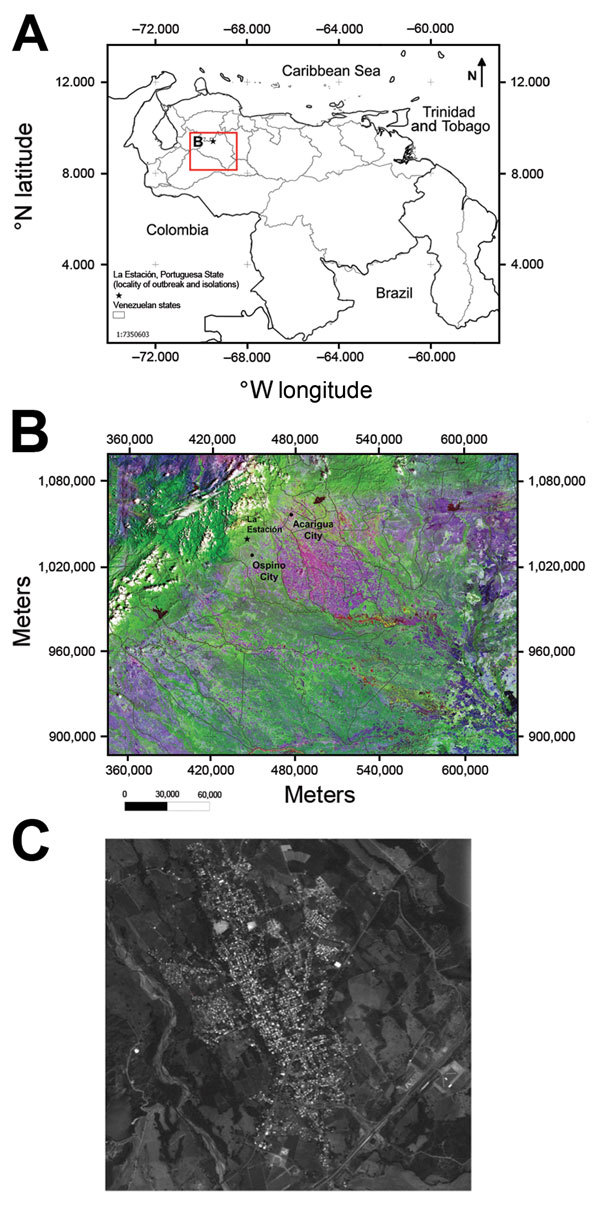
A) Location (red box) of outbreak of Mayaro virus, La Estación village, municipality of Ospino, Portuguesa State, Venezuela, 2010. Scale bar is at the lower left. B) Landsat image of eastern Andes and plains (Llanos) showing topography in Portuguesa State, Ospino, and La Estación, 28.5-m scale (http://glcf.umd.edu/data/landsat/). C) Spot image TM-5, 2.5-m scale, from La Estación, showing forest areas surrounding the urban–rural village (http://www.fii.gob.ve/proyectsFlags.html?value = 5).

La Estación village is located at the northwestern corner of the municipality of Ospino, within Portuguesa State, Venezuela (Figure 1). It is a rural village with a population of 9,538 persons (average age 26 years). In the first quarter of 2010, an outbreak of a febrile illness with arthralgic manifestations was detected. Active surveillance was initiated by the Ministry of Health environmental health team.

### Virus Isolation and Identification

Viruses were isolated from human serum samples and identified on Vero E6 cell monolayers as described (*16*) by using a panel of polyclonal antibodies against alphaviruses and flaviviruses, followed by a MAYV-specific monoclonal antibody (MIAF TRVL4675). Six viruses were isolated from acute-phase serum samples of symptomatic patients. Signs and symptoms and other patient details are described in the results.

### Virus Propagation, Reverse Transcription PCR Amplification, and Sequencing

The 6 virus isolates from Venezuela were sequenced by using the Illumina HiSeq1000 platform (Illumina Inc., San Diego, CA, USA) as described (*24*). To facilitate a more detailed phylogenetic comparison, we determined complete genome sequences for 21 additional MAYV isolates from the World Reference Center for Emerging Viruses and Arboviruses Collection at the University of Texas Medical Branch (Galveston, TX, USA) (Table 1). Viruses were propagated once in Vero cells, and RNA was extracted from cell culture supernatant by using TriZol LS (Life Technologies, Carlsbad, CA, USA) according to the manufacturer’s recommendations. Complete genome sequences were obtained by using reverse transcription PCR (RT-PCR) amplification and sequencing of 6 overlapping RT-PCR amplicons (primer sequences available upon request). RT-PCRs were performed by using the Titan One-Step RT-PCR Kit (Roche Diagnostics, Indianapolis, IN, USA). PCR amplicons were visualized, excised, purified, and sequenced as described (*25*). Sequences were submitted to GenBank under accession nos. KP842794–KP842820.

**Table 1:** Characteristics of Mayaro virus strains sequenced, Venezuela, 2010*

Isolate ID code	Source	Location	Year of collection	Genotype	GenBank accession no.
11A	Human	La Estación, Portuguesa, Venezuela	2010	D	KP842795
12A	Human	La Estación, Portuguesa, Venezuela	2010	D	KP842796
13A	Human	La Estación, Portuguesa, Venezuela	2010	D	KP842797
14A	Human	La Estación, Portuguesa, Venezuela	2010	D	KP842798
15A	Human	La Estación, Portuguesa, Venezuela	2010	D	KP842799
16A	Human	La Estación, Portuguesa, Venezuela	2010	D	KP842794
Ohio	Human	Loreto, Peru	1996	D	KP842807
TRVL15337	Mosquito	Trinidad	1957	D	KP842810
BeH343148	Human	Para, Brazil	1978	D	KP842803
BeH186258	Human	Brazil	1970	D	KP842809
IQU3056	Human	Loreto, Peru	2000	D	KP842808
FSB1131	Human	Bolivia	2006	D	KP842806
IQE2777	Human	Loreto, Peru	2006	D	KP842801
BeAn337622	Monkey	Para, Brazil	1978	D	KP842804
FSB0319	Human	Bolivia	2002	D	KP842805
ARV0565	Human	San Martin, Peru	1995	D	KP842800
BeAn343102	Monkey	Para, Brazil	1978	D	KP842802
FMD0641	Human	Puerto Maldonado, Peru	2005	D	KP842811
FPY0046	Human	Yurimaguas, Peru	2011	D	KP842813
FVB0112	Human	Bolivia	2006	D	KP842814
FPI1761	Human	Iquitos, Peru	2011	D	KP842815
FPI0179	Human	Iquitos, Peru	2011	D	KP842816
FVB0069	Human	Bolivia	2006	D	KP842817
FMD3213	Human	Puerto Maldonado, Peru	2010	N	KP842812
BeH256	Human	Para, Brazil	1955	L	KP842819
BeAr30853	Tick	Para, Brazil	1961	L	KP842820
BeAr505411	Mosquito	Para, Brazil	1991	L	KP842818

*ID, identification.

### Sequence Analysis

Nucleotide sequences were aligned with MAYV sequences available in GenBank by using Clustal X (http://bips.u-strasbg.fr/fr/Documentation/ClustalX/), and manually adjusted by using Se-Al (http://tree.bio.ed.ac.uk/software/seal/). Twenty-nine genomic sequences (i.e., 27 determined in this study and 2 obtained from GenBank) were manually aligned, and untranslated terminal sequences were removed. A second dataset consisting of all partial E2–E1 envelope glycoprotein sequences (n = 68) was also analyzed. All sequences were confirmed as being nonrecombinant by using Recombination Detection Program version 4 (*26*).

The presence and nature of selective pressures acting on the MAYV genome were assessed by using methods available in Datamonkey (*27*), including the single-likelihood ancestor counting (SLAC) and internal fixed effects likelihood (IFEL) methods. Positive and negative selection events at each codon were determined.

### Phylogenetic Analysis

Maximum-likelihood (ML) phylogenetic trees were constructed by using the best-fit general time reversible + gamma 4 + invariable sites model, which was identified by using MODELTEST version 3.7 (*28*). Bootstrapping was performed to assess robustness of topologies by using 1,000 replicate neighbor-joining trees under the ML substitution model. Analyses were performed with PAUP* version 4.0b (Sinauer Associates, Inc., Sunderland, MA, USA).

### Coalescent Analysis

Bayesian coalescent analyses were performed by using a general time reversible + gamma 4 nucleotide substitution model, an uncorrelated lognormal molecular clock model, and a Bayesian Skyline population growth model. To ensure statistical efficiency, we applied a Bayesian stochastic search variable selection procedure (*29*). Inferences were obtained by using a Bayesian Markov chain Monte Carlo approach (*30*) run for 100 million generations with a 10% burn-in period and sampling every 10,000 states. Tracer version 1.5 (http://tree.bio.ed.ac.uk/software/tracer/) was used to monitor stationarity and efficient mixing. TreeAnnotator version 1.8.0 (http://beast.bio.ed.ac.uk) was used to summarize the posterior tree distribution, and FigTree version 1.3.1 (http://beast.bio.ed.ac.uk) was used to visualize the annotated maximum clade credibility (MCC) tree.

## Results

### Outbreak Investigation and Virus Isolation

During January–June 4, 2010, a total of 77 clinical cases compatible with MAYV infection were reported from La Estación. Fifty (65%) were in female patients and 27 (35%) in male patients; >50% of patients were homemakers and laborers, and 38 (49%) were 25–54 years of age (*31*). MAYV was isolated from 6 of 19 acute-phase serum samples, all obtained from symptomatic case-patients. Signs and symptoms for these 6 patients (3 male patients and 3 female patients; age range 15–73 years) are shown in Table 2. Although all 6 patients had arthralgia, several did not have all characteristic signs and symptoms, such as headache (3/6) and nausea and vomiting (4/6). One case-patient (a 73-year-old woman) did not have fever.

**Table 2:** Characteristics of 6 Mayaro virus–infected patients, Ospino, Venezuela, 2012*

Strain ID code	Patient no.	Age, y/sex	Onset date†	Sampling date	Signs and symptoms								
					Fever	Headache	Arthralgia	Nausea	Chills	Nasal congestion	Sore throat	Rash	Cough
11A	1	73/F	Feb 12	Feb 14	No	No	Yes	No	No	No	No	No	No
12A	2	38/F	Feb 12	Feb 14	Yes	Yes	Yes	Yes	No	No	No	No	No
13A	3	19/M	Feb 15	Feb 19	Yes	Yes	Yes	Yes	No	Yes	Yes	No	Yes
14A	4	46/F	Feb 18	Feb 19	Yes	No	Yes	No	Yes	Yes	Yes	Yes	No
15A	5	15/M	Feb 28	Mar 1	Yes	Yes	Yes	No	No	No	No	No	Yes
16A	6	47/M	Mar 13	Mar 16	Yes	No	Yes	No	Yes	No	No	No	No

*ID, identification. †Start date of signs and symptoms.

### Sequence Divergence and Phylogenetic Analysis

In addition to sequencing the complete genomes of the 6 outbreak strains from Venezuela, we also sequenced complete genomes for 21 strains representing the full spectrum of genetic diversity, and known temporal and spatial distributions of MAYV (Table 1). Percentage nucleotide and amino acid sequence identities relative to outbreak strain 16A across all 9 genes of the genome for 10 strains that represent the full spectrum of genetic diversity determined for MAYV in this study are shown in Table 3. Analysis of nucleotide and amino acid sequence identities showed that MAYV is highly conserved (nucleotide sequence identities 96.4%–100% and amino acid sequence identities 97.7%–100%) among genotype D strains (Table 3). Comparison of genotype L with genotype D showed greater divergence (nucleotide sequence identities 83.8%–88.6% and amino acid sequence identities 90.9%–97.4%) for all genes.

**Table 3:** Nucleotide and amino acid sequence identities among 9 major genes of the MAYV VZ2010 outbreak strain and 10 representative MAYV strains, Venezuela, 2010*

MAYV VZ2010 gene	Strain

Genotype D									Genotype L	
Ohio	FSB 1131	ARV 0565	BeAn 337622	MAYLC	BeH 186258	TRVL 15537	FMD 3213		BeH 256	BeAr 505411
*nsp1*	99.4 (99.1)	98.9 (99.1)	98.6 (99.1)	98.1 (98.7)	98.4 (98.9)	97.6 (99.1)	97.3 (98.1)	95.6 (98.7)		88.6 (97.0)	88.6 (97.0)
*nsp2*	99.5 (99.7)	98.7 (99.5)	98.6 (99.6)	98.4 (99.5)	98.1 (99.1)	96.8 (99.1)	96.9 (98.9)	93.7 (99.0)		86.2 (97.4)	86.2 (97.4)
*nsp3*	99.7 (100.0)	98.6 (100.0)	97.8 (99.0)	98.1 (99.2)	97.7 (98.8)	96.2 (98.5)	96.7 (98.3)	93.3 (78.4)		82.7 (76.9)	82.5 (76.9)
*nsp4*	99.2 (100.0)	98.3 (99.7)	98 (99.8)	97.8 (99.8)	97.5 (99.8)	97.1 (99.3)	97.1 (99.2)	92.5 (98.2)		86 (97.2)	86.2 (97.2)
C	99 (100.0)	98.7 (99.6)	98.7 (100.0)	98.1 (99.6)	97.9 (100.0)	96.5 (99.6)	97.0 (100.0)	93.5 (98.8)		87.1 (96.1)	88 (96.9)
E3	99.5 (100)	98.5 (100.0)	96.5 (100.0)	97 (100.0)	96.5 (100.0)	97.5 (100.0)	97.0 (100.0)	93.4 (98.5)		83.8 (90.9)	84.8 (90.9)
E2	99.3 (99.8)	98.4 (99.8)	97.9 (99.1)	98.3 (100.0)	97.5 (99.5)	96.1 (99.8)	96.4 (98.8)	93.3 (98.8)		86.3 (97.4)	86.2 (96.9)
6K	100 (100.0)	98.3 (98.3)	98.9 (100.0)	98.3 (100)	98.9 (100.0)	98.3 (100.0)	97.8 (100.0)	97.8 (100.0)		87.8 (95.0)	88.3 (96.7)
E1	99.5 (99.8)	99.1 (99.8)	98.7 (99.5)	98.5 (99.8)	98.4 (99.8)	97.9 (99.3)	97.6 (97.7)	94.3 (98.4)		88 (96.1)	87.6 (96.3)

*Values in parentheses are amino acid identities. MAYV, Mayaro virus; *nsp*, nonstructural protein; C, capsid; E3, envelope small polypeptide; E2, envelope glycoprotein 2; 6K, envelope small polypeptide; E1, envelope glycoprotein 1.

**Figure 2:**
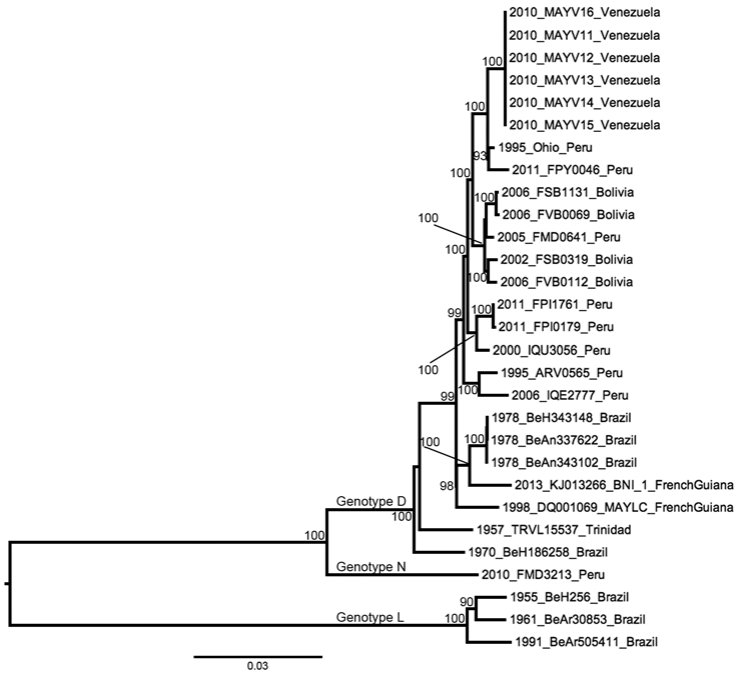
Midpoint-rooted maximum-likelihood phylogeny of 29 Mayaro virus strains on the basis of complete genome sequences, Venezuela, 2010. Nodes are labeled with bootstrap values ≥90%. Tip labels indicate year of isolation, strain name, and country of isolation. Scale bar indicates percentage nucleotide sequence divergence. Isolates DQ001069 and KJ013266 were previously sequenced and obtained from GenBank.

**Figure 3:**
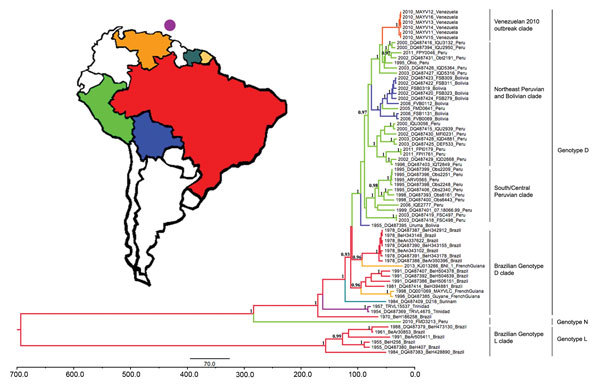
Bayesian maximum clade credibility tree for Mayaro virus in the Americas on the basis of 1,731 nt of envelope (E2–E1) genes, Venezuela, 2010. The purple circle above Venezuela indicates the island of Trinidad. Taxon labels indicate year of isolation, strain designation, and country of isolation. Terminal branches of the tree are colored according to the sampled location of the taxon at the tip. Internal branches are colored according to the most probable (modal) location of their parental nodes. Nodes with posterior probabilities (clade credibilities) ≥0.90 are indicated in black. Scale bar indicates time in years.

An ML phylogeny based on the complete genome sequences of 29 MAYV strains and with a topology similar to that reported by Powers et al. (*6*) for partial E2–E1 sequences is shown in Figure 2. These results suggested that the trees based on partial genomes are sufficiently resolved for detailed coalescent analyses. A Bayesian MCC tree based on the partial E2–E1 fragment (nt 9,412–11,139 for DQ001069) from 68 sequenced MAYV strains, and with posterior probabilities indicated at relevant nodes is shown in Figure 3. The color of each lineage represents the most probable geographic location for the hypothetical ancestor at the node representing it. This phylogeny was consistent with the complete genome tree (Figure 2) and the phylogeny of Powers et al. (*6*). In addition to the previously described distinction between genotype D and L strains, there was some evidence of geographic structure within genotype D (Figure 3). In both our ML and MCC phylogenies, the 2010 outbreak sequences from Venezuela grouped within genotype D (posterior probability >0.99), and were most closely related to strains from Peru.

In addition to the previously described genotypes, a recently isolated (2010) strain from Peru (FMD3213) was intermediate between genotypes D and L and showed strong statistical support in both phylogenies. Given its year of isolation, strong support for its position in both phylogenies, and intermediary genetic distance from both genotypes D and L (Table 3), this strain might represent a previously undetected genotype, which we designate N.

### Selection Analysis

A mean global nonsynonymous:synonymous (dN:dS) ratio of 0.057 calculated by using the SLAC algorithm indicated that, similar to other arboviruses, purifying selection is the predominant evolutionary force driving MAYV evolution. This result was supported by the 274 codons found by SLAC and the 539 sites detected by IFEL to be under purifying selection. One codon was identified as being under positive selection by using SLAC and 5 by using IFEL (p<0.1). None of the 5 sites detected by IFEL delineated the 2010 outbreak strains, but these 5 sites instead primarily defined genotype L.

### Evolutionary Rates and Dates of Divergence

The mean evolutionary rate estimated from the genomic MAYV dataset was 1.67 × 10^−4^ substitutions/site/year (95% highest posterior density [HPD] 1.02 × 10^−4^–2.41 × 10^−4^). The rate for the clade containing the 2010 outbreak strains from Venezuela was estimated to be 1.34 × 10^−4^ substitutions/site/year (95% HPD 0.8 × 10^−4^–1.9 × 10^−4^) for partial E2–E1 sequences and 1.57 × 10^−4^ substitutions/site/year (95% HPD 1.1 × 10^−4^–2.1 × 10^−4^) for complete genome sequences. Rates for other MAYV lineages were 0.87 × 10^−4^–2.35 × 10^−4^ substitutions/site/year for complete genome sequences and 0.75 × 10^−4^–2.40 × 10^−4^ substitutions/site/year for partial E2–E1 sequences.

Estimated dates of divergence for selected lineages are shown in Table 4. The most recent common ancestor (MRCA) was estimated to be 1864 (95% HPD 1822–1904) for genotype D and 1877 (95% HPD 1836–1915) for genotype L on the basis of partial E2–E1 data. All dates estimated within genotype D were strongly supported by the results based on complete genomes (Table 4). For example, the MRCA for 2010 isolates from Venezuela was estimated to have occurred during 2003–2009 (95% HPD) and have a mean estimate of 2007 based on the partial sequences. The estimate based on complete genomes was 2008 and had a smaller 95% HPD (2007–2009). The 95% HPDs for the genotype L time of MRCA based on partial E2–E1 sequences were wider. The MRCA for the node containing genotypes N and D was estimated to be 1657–1833 (95% HPD).

**Table 4:** Estimated dates of divergence derived from partial E2 and complete genome sequences for major Mayaro virus lineages, Venezuela, 2010*

Lineage	Date for time of MRCA for partial E2–E1 sequences (95% HPD)	Date for time of MRCA for complete genome sequences (95% HPD)
Venezuelan outbreak strains	2007 (2003–2009)	2008 (2007–2009)
Northeast Peruvian and Bolivian clade	1952 (1935–1967)	1966 (1940–1970)
South/Central Peruvian clade	1968 (1953–1980)	1977 (1967–1987)
Brazilian genotype D clade	1864 (1822–1904)	1872 (1836–1906)
Brazilian genotype L clade	1877 (1836–1915)	1905 (1882–1926)
Peruvian genotype N clade	1750 (1657–1833)	1746 (1658–1823)

*E, envelope; MRCA, most recent common ancestor; HPD, highest posterior density.

## Discussion

MAYV is a major emerging pathogen in northern South America and causes sporadic outbreaks of arthralgic disease in the Brazilian Amazon and eastern Bolivia. To date, these outbreaks have been relatively small, except for the epidemic in Belterra, Brazil, in 1977–1978. However, antibody detection and virus isolation rates indicate that MAYV commonly infects persons residing near enzootic transmission foci, and high incidence rates have been detected by using clinical surveillance (*32*).

The Mayaro fever outbreak at La Estación in 2010 is noteworthy because, with the exception of a family found to be seropositive (*20*), it probably represents the first outbreak documented in Venezuela. Also, with 77 reported cases, it is one of the largest outbreaks ever described. The signs and symptoms recorded corresponded to an influenza-like illness with arthralgia in most cases. One case-patient, a woman who was confirmed to be MAYV positive, had persistent arthralgia 1 month after infection. Cases of Mayaro fever are grossly underestimated in South America because extensive overlap in signs and symptoms means they typically fall under the dengue umbrella. It is essential that countries in South America test for MAYV when patients have dengue-like illness that also involves arthralgia to determine the true incidence of MAYV infection.

A larger proportion (65%) of cases were in female patients during this outbreak. Previous studies in Brazil and Bolivia have shown no sex bias during outbreaks of Mayaro fever (*9**,**10*), but a recent clinic-based surveillance study in Bolivia and Peru demonstrated that male patients were more likely to be MAYV infected, probably because of occupational exposure (*32*). It is unclear why we observed the opposite sex bias during the outbreak in La Estación. Further information on occupational exposure at this location is necessary to better understand the demographics of this outbreak of Mayaro fever.

La Estación is located in a former tropical forest that has recently been converted for coffee production and other farming (*33*). Several monkey species (i.e., *Cebus olivaceus* and *Alouatta seniculus*) and competent MAYV vectors (i.e., *Haemagogus* mosquitoes) are present within this village (*33**,**34*), which suggests enzootic circulation near human residences and work locations, possibly enhanced by encroachment into forests by local residents. This conclusion is supported by our data, which indicate that 50% of female case-patients, including those with higher antibody titers, primarily performed home activities; 37.5% of seropositive male patients performed coffee agricultural activities; and 63% of all seropositive persons resided near the coffee plantation.

Nonsynonymous mutations that defined a specific group, clade, or lineage were identified manually. Uninformative mutations were not counted; only synapomorphies that were unique to a group or cluster were noted. There were 143 nonsynonymous synapomorphic mutations of interest, of which 114 were unique to genotype L. The 5 amino acid positions that were detected to be under positive selection and to delineate the genotype L strains were Leu→Ala/Val at position 518 in NSP1; Ala→Prol and Val→Thr at positions 298 and 386 in NSP3, respectively; Ala→Lys at position 249 in NSP4; and Leu→Thr at position 300 in the E1 protein.

In addition, strains from the outbreak in Venezuela in 2010 were also defined by 5 nonsynonymous mutations: Ser→Gly and Pro→Leu at positions 487 and 523 in NSP1, respectively; Val→Ile and His→Tyr at position 586 and 665 in NSP2, respectively; and Ile→Leu at position 156 in the E1 protein. However, these mutations were not identified as being subject to positive selection. Also, none of these unique mutations is in a genomic region known to affect the virulence or transmissibility of alphaviruses, and these amino acid substitutions are mostly conservative in nature. Whether these substitutions played any role in the emergence of MAYV is unclear. Reverse genetic studies are needed to determine if any of these substitutions cause major phenotypic changes.

In addition to the previously described MAYV genotypes (*6*), we detected a new genotype N that is genetically distinct and has strong phylogenetic support. We also further delineated several clades that segregated by geographic region (Figure 3). The estimate for the origin of MAYV strains in South America (i.e., 670 years [95% HPD 441–912 years] before 2013) should be interpreted with caution because a recent study of Venezuelan equine encephalitis complex alphaviruses showed that time of MRCA estimates for ancestral nodes dating more than a few hundred years ago is influenced by internal branch compression resulting from strong purifying selection (N.L. Forrester et al., unpub. data). Our coalescent analyses also estimated that genotypes D and L diverged ≈107–150 years before 2013, which suggests a relatively recent origin for these genotypes.

The apparent restriction of genotype L to Brazil, when compared with the wider distribution of genotype D, suggests geographic constraints on MAYV dispersal within Brazil. Because there is no apparent geographic barrier that can account for this distribution, the lack of genotype L strains from other countries might represent sampling bias, rather than a true population subdivision. However, we cannot exclude the possibility that potential restrictions associated with vector competence, vector distributions, or alternative vertebrate amplification hosts might affect this apparent population subdivision. The 6 virus sequences from the outbreak in Venezuela in 2010 (the only representatives from Venezuela) grouped as a monophyletic clade within genotype D and showed strong support as determined by genomic and partial sequences. Because genotype D strains show genetic diversity derived predominantly from viruses in Peru, it is not surprising that strains from Peru occupied positions basal to strains from the outbreak in Venezuela in 2010. Although there was strong support for the ancestral strain to have been derived from strains in Peru in 2010, this finding might also have resulted from sampling bias. There is a dearth of MAYV isolates and sequences from several intervening countries, including Brazil, Ecuador, and Colombia, and recent virus sequences from these countries are needed to identify whether MAYV is maintained continuously in Venezuela or if virus spread influences emergence. For example, Ecuador and Colombia might form the ecologic bridge between strains derived from Venezuela and the ancestral strains from Peru. Persons with antibodies against MAYV have been reported among Ecuadorian soldiers living in the Amazon (*18*), which supports this hypothesis.

Recent work on alphaviruses from Venezuela showed that a 1999 MAYV sequence grouped most closely with sequences from Trinidad in the Brazilian genotype D clade (*35*). However, these results were based only on E1 3′-untranslated region sequences, and their sequences could not be included in our analyses. Given the position of the 2010 sequences from Venezuela in our phylogeny, it appears that the 2010 outbreak strains did not descend from MAYV isolates previously circulating in Venezuela (i.e., at least since 1999). However, it is also possible that there is co-circulation or regionally independent evolution of genetically distinct MAYV strains within Venezuela. Yellow fever virus has a similar enzootic transmission cycle and was recently shown to undergo regionally independent evolution in Venezuela (*36*), which supports this hypothesis.

Analysis of sequence identities among MAYV genes demonstrated a high degree of conservation, similar to that seen for North American eastern equine encephalitis virus (*37*) and western equine encephalitis virus (*38*). These alphaviruses have relatively recent estimated times of MRCAs, occupy specific and similar ecologic niches, and appear to undergo continuous evolution without major subdivision by geography or time. The MAYV tree topologies we observed also suggested continuous circulation within a distinct ecologic niche in South America. Eastern and Western equine encephalitis viruses have avian hosts, which might account for the lack of population subdivision by geography. In contrast, yellow fever virus, which is maintained by nonhuman primates, has a more geographically structured phylogeny (*39*). Despite the observation of high seroprevalence in nonhuman primates and isolation of MAYV from primatophilic mosquitoes (*6**,**10*), our tree topology and genetic conservation in MAYV suggest that birds or other highly mobile hosts might play some role in its dispersal. This finding could account for reduced geographic structure and observed branching patterns in MAYV phylogeny. Previous work has shown that MAYV replicates efficiently in avian cell cultures, such as Peking duck kidney cells and chick embryonic fibroblasts (*40*), and can achieve titers as high as 5.7–7 logs, suggesting that birds could be suitable reservoir hosts. However, further studies are necessary to determine if MAYV can replicate at the increased temperatures (≈43°C) in birds during levels of high activity. We hypothesize that during epizootics/epidemics, nonhuman primates are spillover hosts that might be especially susceptible to MAYV infection. The inability to continuously isolate or detect MAYV from *Haemagogus* spp. mosquitoes and nonhuman primate hosts in disease-endemic areas is consistent with the hypothesis that MAYV is maintained in a transmission cycle involving other vertebrate reservoir hosts.

This outbreak in Venezuela indicates that MAYV is a major emerging pathogen in South America, where persons residing near enzootic transmission foci might be at increased risk because of anthropogenic incursions. Strains from the outbreak in Venezuela in 2010 belong to genotype D and are distinct in the MAYV phylogeny. The single isolate from Puerto Maldonado in the Madre de Dios region of Peru in 2010 represents the only genotype N strain. Further surveillance at this and neighboring locations would be useful to determine the true extent of the genetic diversity of genotype N. Filling the current gaps in sequence data for geographic and temporal distributions of MAYV is needed to increase the phylogenetic resolution and aid our understanding of the evolution and spread of this emerging arthralgic alphavirus in the Americas.
